# Microscopy with microfluidics in microgravity using FlightScope

**DOI:** 10.1038/s41526-025-00470-3

**Published:** 2025-05-06

**Authors:** Thomas Wareing, Alexander Stokes, Katrina E. Crompton, Koren Murphy, Jack Dawson, Yusuf Furkan Ugurluoglu, Connor Richardson, Hongquan Li, Manu Prakash, Adam J. M. Wollman

**Affiliations:** 1 School of Engineering, Newcastle University, Newcastle-Upon-Tyne, NE2 4HH UK; 2 Newcastle University Biosciences Institute, Newcastle-Upon-Tyne, NE2 4HH UK; 3 Stanford University, Stanford, CA 94305 USA

**Keywords:** Cell biology, Microbiology

## Abstract

With planned missions to the moon and Mars, it has never been more important to study the impact of microgravity on biological organisms. Parabolic flights are one of the most accessible microgravity research platforms but present challenges: short periods of microgravity and aircraft vibration. Live-imaging is necessary to readout any real-time phenotypes so we developed FlightScope, a new microscopy and microfluidics platform to study dynamic cellular processes in microgravity.

Understanding the impact of altered gravity on living organisms has never been more important. Planned future missions to land humans back on the moon, and Mars, will require extended periods of time in altered gravity. It would take at least 7–9 months to travel to Mars^1^. This time in microgravity has an adverse effect on astronaut health, including well characterised impacts on bone density^1^ but also less explored impacts on molecular and cellular processes in the immune system^2–6^ and metabolism^3,5–12^. Extended durations in space will also require new technologies in self-sufficiency and miniaturisation due to the cost of carrying cargo into space. Synthetic biology, where micro-organisms are harnessed to grow foods and medicines, is an attractive technology for space travel as it requires only minimal starting material to be carried which can be scaled up in bioreactors. Baker’s yeast is a key organism for synthetic biology. Yeast has been engineered to produce medicines: morphine^13^, penicillin^14^, and breviscapine, a drug for the treatment of angina^15^. Yeast has also been used to produce haemoglobin for cultured meat^16^. Additionally, yeast is a key model organism for studying basic biological processes also happening in human cells^17^.

There are many different platforms for simulated and real microgravity that can be used for research. Drop towers allow the least amount of microgravity experiment time with an average of only 6–10 s^18,19^. Sounding rockets allow 3–15 min of microgravity^20^ but are also costly^21^. Neither of these microgravity platforms allow for operators to perform their experiments directly. The International Space Station (ISS) offers the longest period of reduced gravity but is difficult to access and costly. Parabolic flights in specially fitted jet aircraft offer 15–20 s of microgravity per parabola and are therefore one of the most accessible and cost-effective microgravity platforms currently. Parabolic flights also allow for dynamic experiments whereby the effects of changes in effective gravity can be observed in real time and possible adjustments made accordingly.

The microscope has been a core tool for biology research for over 300 years^22^, including in microgravity research (summarised in Table 1). Several microscopes have been used in altered gravity environments, including for permanent use on the International Space Station (ISS). These include the Bioserve Microscopy Platform (BSMP)^23^, the Light Microscopy Module (LMM)^4^ and the Mochii ISS National Laboratory (NL), the first scanning electron microscope capable of providing high-resolution images on the ISS^24^. The FLUMIAS-DEA fluorescence microscope has also been on board the ISS Space Tango Facility temporarily and used to image human macrophage cells^10^. Microscopes have also been used on the space shuttle, including the NIZEMI which used a commercial Zeiss microscope to study the behaviour of *Paramecium biaurelia* in response to gravity changes^2^. CODAG, a stereo long-distance microscope, equipped with high-speed CCD cameras was used to image dust particle motion and the structure of dust aggregates of sand flown onboard a space shuttle^3^. On parabolic flights, commercial brightfield microscopes have been used to image migrating immune cells^6^ and, mounted on a silicone damped rack, the beating pattern of algal flagella^5^. Bespoke microscopes like the confocal laser spinning disc microscope, Fluorescence Microscopy Analysis System (FLUMIAS), and the digital holographic microscopy system, (DHM), have also been used on parabolic flights to image cytoskeletal changes in human follicular thyroid carcinoma cells^10^ and mouse myoblast cells^8^ respectively. The Mars Rover too has its own magnifying hand lens, MAHLI (Mars Hand Lens Imager), that is used to take microscopic images of minerals and structures in Mars rocks and soil^25^. The Mars hand lens only uses a single lens as compared to a compound microscope which uses multiple. Of these microscopes, none have incorporated a microfluidic system to allow live, real-time observations of changes to sample behaviour during changes to its chemical environment. Microfluidics is a key tool in yeast^17,26,27^ and synthetic biology research^28–30^ and is an extremely useful technology for microgravity platforms which offer very little time to activate and record changes to biological behaviour in samples.

**Table 1:** Summary of previous 0g microscopes

	Year	0g platform	Microscope type	Attributes
NIZEMI^2^	1994	Space Shuttle Columbia, IML-2	Commercial (Zeiss)	Brightfield and Darkfield imaging Thermal control of samples (14-38°C) Magnification 1.25x-40x, estimated resolution 1.4–0.3 µm
CODAG^3^	1998	STS-95	Commercial	Stereo brightfield Reported field of view 0.25 ×0.25 mm^2^, spatial resolution of 1 µm, depth of field of ~80 µm, temporal resolution of 5 ms
LMM^4^	2000+	ISS	Bespoke, modified Leica DM-RXA	Brightfield, Darkfield, DIC and fluorescence imaging Other capability: Dynamic and static light scattering, Spectrophotometry, Optical tweezers Magnification 10x-100x (oil), estimated resolution 0.6–0.2 µm
BiozeroBZ-8000^5^	2006	ZeroG Parabolic Flight	Commercial (Keyence)	Brightfield imaging with fluorescence capability Magnification 10x-100x (oil), estimated resolution 0.6–0.2 µm
BioLab^41^	2008	ISS	Bespoke	Brightfield, Darkfield and phase contrast imaging Combined sample incubation Magnification 40x, Low resolution mode: 1.6 µm/1 mm field of view High resolution: 0.6 µm/0.25 mm field of view
ESA PFC 2008^6^	2009	Zero-G Parabolic Flight	Commercial (Leica DMIL)	Brightfield and fluorescence Magnification not reported
DHM-RPM^7^	2010	Random Positioning Machine (RPM)	Bespoke	Digital holographic microscopy including off-axis imaging Reported field-of-view 65×65 μm^2^ on a 512×512-pixel region lateral resolution in the 500 nm range
DHM-RPM-PFC^8^	2010	RPM ZeroG Parabolic Flight	Bespoke	Digital holographic microscopy including off-axis imaging combined with LED fluorescence (470 nm excitation) Magnification 60x, estimated resolution 0.3 µm lateral resolution in the 500 nm range^7^
DHM-SM^9^	2012	SM, JASTEC	Bespoke	Digital holographic microscopy including off-axis imaging Magnification 60x, resolution not given
MAHLI^25^	2012	Mars Rover	Bespoke	Portable, automatic, white and UV light capabilities. 2-megapixel colour camera Resolution of ∼30 μm per pixel
FLUMIAS^10^	2016	Zero-G Parabolic Flight TEXUS 52 Sounding Rocket	Bespoke	Confocal laser spinning disc imaging Temperature-controlled fixation unit 3 diode lasers for fluorescence (405 nm/120 mW, 488 nm/200 mW and 642 nm/140 mW) One diode-pumped solid state (DPSS) laser (561 nm/150 mW) Reported axial resolution ~1.5 μm Magnification 40x, estimated resolution 0.3 µm
BioServe Microscopy Platform (BSMP) [3]	2016	ISS	Bespoke	Brightfield microscopy Magnification not reported
FLUMIAS-DEA^11^	2018	ISS, Space Tango Facility	Bespoke	Capabilities as for FLUMIAS^10^ combined with super-resolution imaging using Structured Illumination Microscopy (SIM), resolution 230 nm
Image acquisition module^39^	2020	RPM-NASA MSSF	Commercial (Dino-Lite)	Brightfield, darkfield and fluorescence imaging Magnification 20x, estimated NA of dinolite ~0.14 giving resolution ~3 µm^42^
MochiiISS-NL^42^	2020	ISS	Commercial (Mochii)	Scanning electron microscope

Resolutions as reported from publication or, where possible, estimated from the numerical aperture (NA) as wavelength/2NA.

We developed FlightScope, a new microscopy and microfluidics platform specifically designed to operate in reduced or altered gravity environments. Flight-Scope is based on the open-source *Simplifying Quantitative Imaging Platform Development and Deployment* (SQUID) microscope^31^, with an adapted design to be resistant to vibration and differing gravitational/inertial forces. The microscope also features fluorescence imaging using excitation with a 470 nm LED. Alongside this, a bespoke five-channel microfluidic pump system was built, designed to operate in low and high gravity, incorporating a bespoke microfluidic chip mounting system to facilitate fast sample changes during flights. We tested the system on board a European Space Agency (ESA) parabolic flight, imaging live yeast, *S. cerevisiae*, in microgravity and hypergravity while injecting fluorescent glucose. Our open-source platform will enable future live cell experiments under microgravity and, due to its rugged design, experiments in extreme environments on earth.

## Design of a vibration resistant microscope

**Fig. 1:**
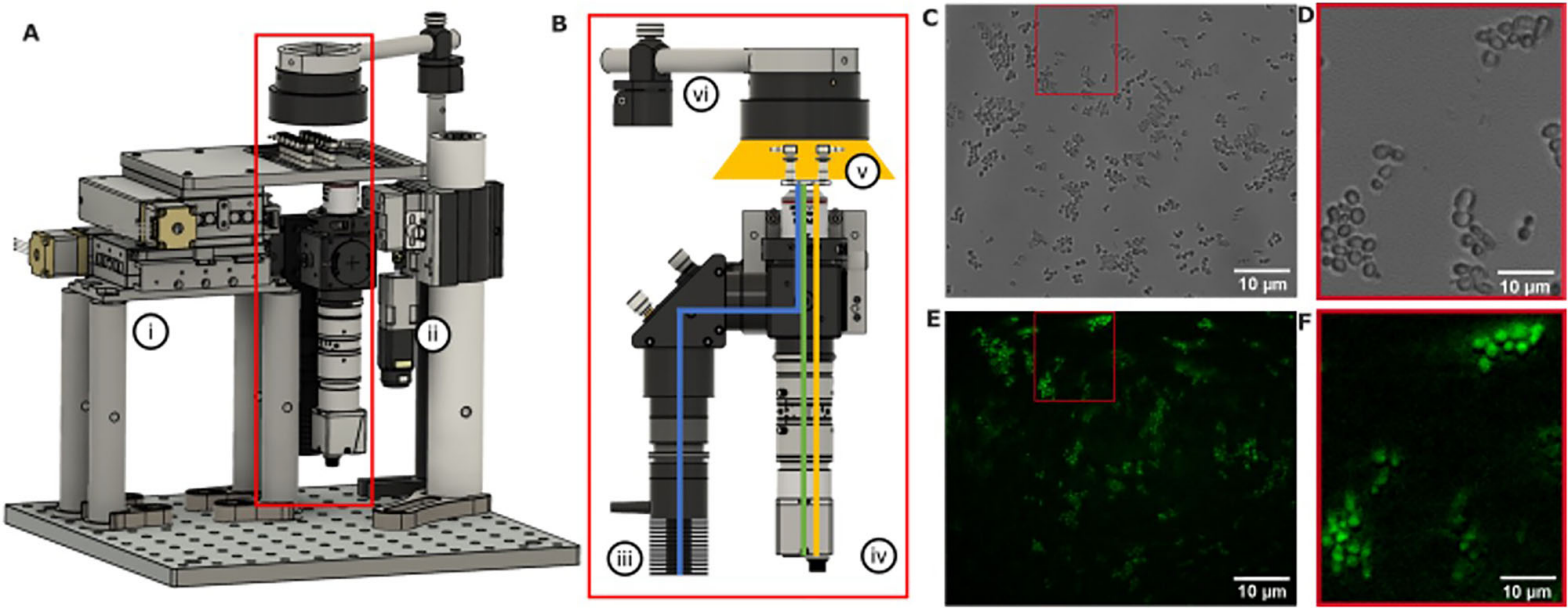
FlightScope microscope. A Schematic of microscope showing (i) motorised xy stage (ii) a central vibration mast which absorbs vibrations felt by the optical train. **B** The microscope optical train is shown in the red outline and consists of (iii) a fluorescence LED, (vi) brightfield LED array, (v) sample and (iv) camera. The blue and green lines depict the excitation and emission optical paths respectively. The yellow line depicts the brightfield optical path. **C** A brightfield image of yeast taken with Flight-Scope with **D** showing a zoomed in region. **E** A fluorescence image of yeast stained with fluorescent glucose 2-NBDG.with **F** showing a zoomed in region. (100 ms exposure, 20% illumination power).

The core of Flight-Scope is a vibration-resistant fluorescence microscope. We designed the microscope based on the open-source SQUID microscope^31^ (Fig.1A). The microscope was capable of two key imaging modes - brightfield and fluorescence. These were implemented using a top-mounted RGB LED pixel matrix to generate white light illumination for brightfield and a Thorlabs 809 mW 470 nm LED for epi-fluorescence. Images were formed using a 40x/0.75 Olympus Plan Fluor objective, a f = 75 mm imaging lens as tube lens and a camera with Sony IMX178 CMOS monochrome sensor. A 40x air objective was chosen in part as higher magnification objects are subject to high vibration sensitivity. Standard microscope slide-based samples were mounted on a bespoke 60-60 mm x-y motorised stage. The microscope was controlled using a joystick and custom SQUID Python-based software^31^ for image-viewing, capture and control of x-y-z motion. The microscope has a field of view of 467 µm × 311 µm (3072 × 2048 pixels) with sample side pixel size of 152 nm. At full resolution the camera supports 60fps. A filter cube allowing excitation from 465 to 495 nm and emission at 515–555 nm was used. The software allowed switching between brightfield and pseudocoloured fluorescence channels during imaging at 0.5 frames/second. Fluorescence illumination intensity could reach 0.05–1 mW/mm^2^ and was controlled by a Thorlabs LEDD1B - T-Cube LED Driver. Vibration resistance was engineered into the microscope by mounting the optical train on a 2” dampened post (Fig. 1Aii) and the experimental setup placed on four dampeners.

**Fig. 2:**
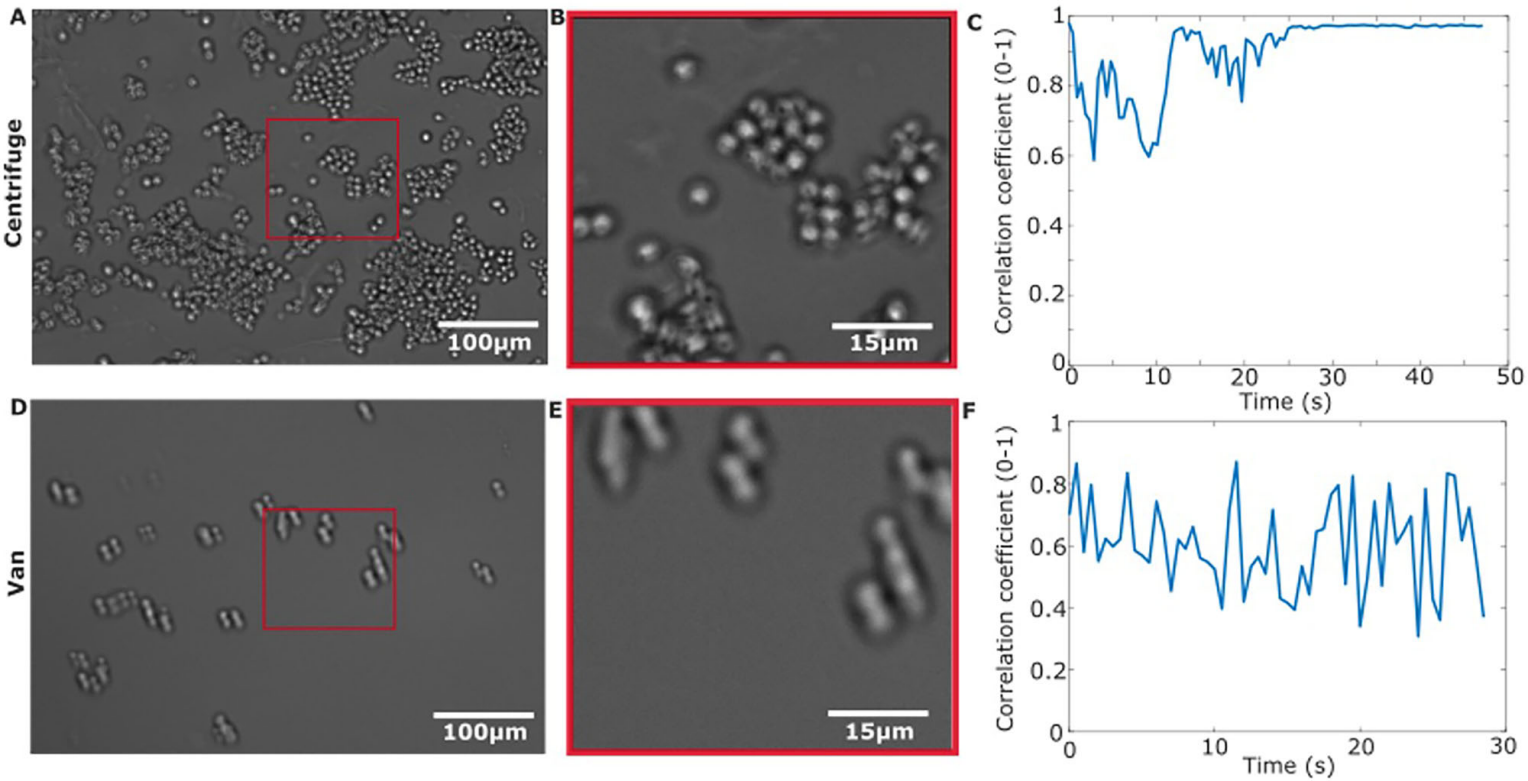
FlightScope vibration testing in the lab. **A** Brightfield image of yeast cells taken while Flight-Scope was mounted on a benchtop centrifuge. **B** A zoomed-in ROI brightfield taken on the centrifuge. **C** Plot of correlation coefficient of cell position in consecutive frames vs time for images taken while the microscope was on the centrifuge. **D** Brightfield image of yeast cells taken while the microscope was mounted inside a van. **E** A zoomed-in ROI brightfield taken on the van. **F** Plot of correlation coefficient of cell position in consecutive frames vs time for images taken while the microscope was mounted inside a van. All images 100 ms exposure.

We tested the imaging capability of the microscope by imaging live yeast, *S. cerevisiae*, in brightfield (Fig. 1C, D) and in fluorescence (with fluorescent glucose analogue 2-NDBG, Fig. 1E, F). We also characterised the vibration resistance of our microscope on the ground by mounting the microscope onto a tuneable vibration source (centrifuge) and imaging live yeast cells (Fig. 2A, B), reaching vibrations of up to 13,000 rpm (217 Hz), similar to the range of vibration onboard a flight^32^. Vibration in the image was quantified by calculating the cross-correlation between consecutive images in MATLAB. We found that we could image yeast cells successfully in these conditions and the correlation only dropped to a minimum of 0.6 (Fig. 2C). We also tested the vibration resistance of the microscope by placing it inside a vehicle – a van - (Fig. 2D, E) with the engine running idly and found greater but tolerable changes in the cross-correlation of the yeast image videos (Fig. 2F).

## Microfluidic system

**Fig. 3:**
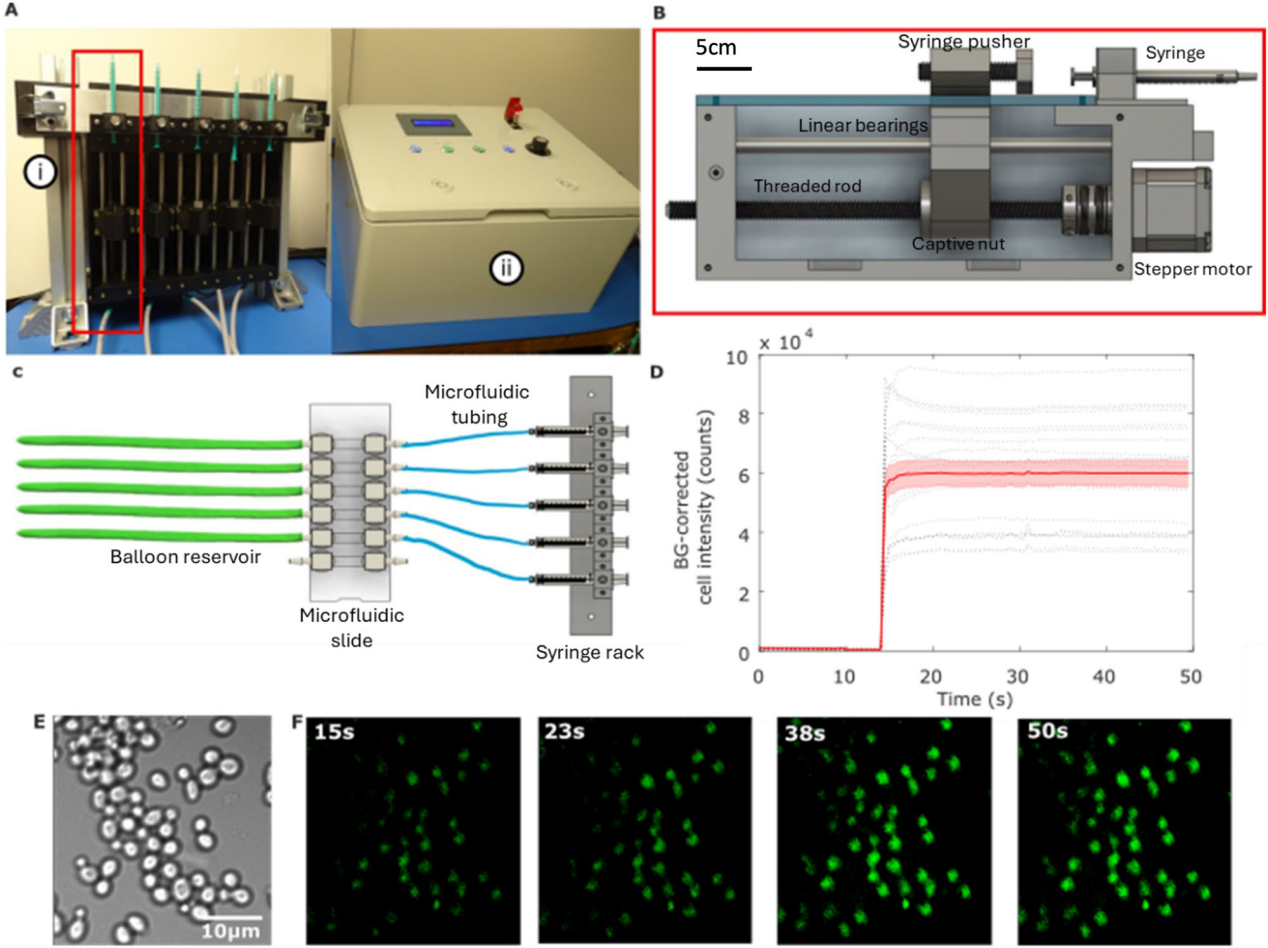
FlightScope microfluidics. **A** The FlightScope syringe pump with clasp-based syringe rack (i) and user interface (ii). **B** Syringe driver schematic. **C** Schematic of panel-mounted sample system with syringe-rack, inlet ports (right) and outlet ports in green (left). Not to scale. **D** Fluorescent 2-NBDG uptake vs. time of yeast cells for 10$\mu$M 2-NBDG concentration *n* = 20 cells, black dotted lines are individual cell traces with their mean (red lined) and standard error (red shading). **E** Brightfield image of yeast taken with the Flight-Scope and **F** fluorescence images taken over a course of 15, 23, 38, 50 s after 2-NBDG injection showing increases in cell intensity over time. (100 ms exposure, 20% illumination power).

We designed a bespoke microfluidic system to allow dynamic changes of the sample fluidic environment during the short gravity changes on board a parabolic flight (Supplementary Fig. S2A-B), designed to operate in altered gravity and with aircraft vibration. The system used stepper motors on a 3D-printed chassis (Fig. 3A, B) to drive 5 syringes with flow rates of 75 μL/s using 1 mL syringes. The system was controlled with a custom user interface (Fig. 3A(i), (ii)) that allowed individual syringe selection, dosing, jogging forward and backward and homing (Supplementary Fig. S4A) pumps for fresh syringes. All 5 syringes could be changed over, thanks to an innovative clasp-based panel design (schematic in Fig. 3C and shown mounted in Fig. 3A(i)), with all 5 syringes mounted to a panel and connected to a microfluidic chip.

We tested the system by imaging the dynamic real-time uptake of fluorescent glucose in yeast cells. We adhered cells using lectin, Concanavalin A, in a microfluidic chamber and pumped in fluorescent glucose analogue, 2-NBDG (Fig. 3D–F). Yeast cells became increasingly fluorescent as they took up glucose (Fig. 3F). We quantified the fluorescence increase due to glucose uptake in each cell using bespoke MATLAB software, FRETzel^33^, observing rapid cellular uptake (Fig. 3D).

## Designing the microscope for operation on a parabolic flight

**Fig. 4:**
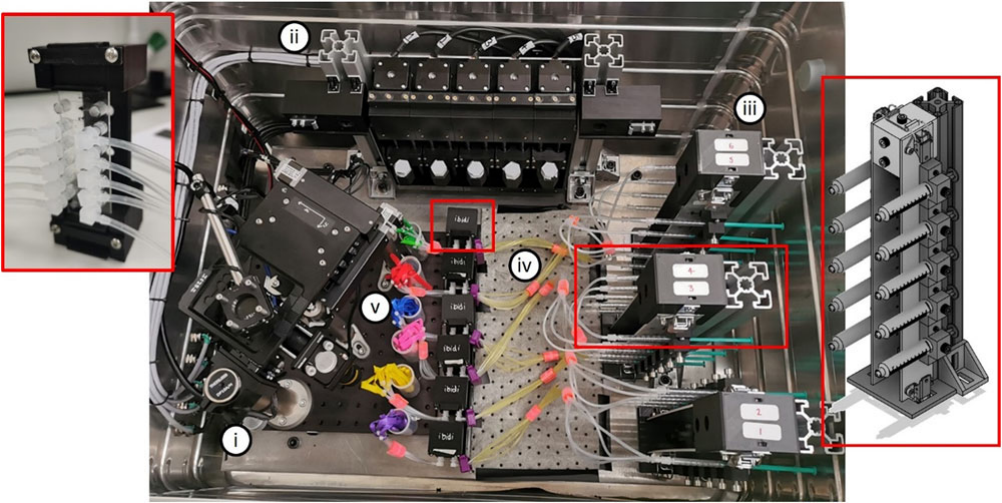
FlightScope microfluidic change-over system. (i) Flight-Scope and (ii) syringe pump inside the Zarges box with (iii) three syringe rack holders, also depicted in the rightmost red outline, each holding a sample set, depicted in the leftmost red outline with (iv) primed glucose inlet tubing and (v) stowed outlet balloons.

We further engineered FlightScope for operation on-board a parabolic flight. The microscope needed to be leak-proof to prevent substances coming in to contact with experimenters during the microgravity portions of the flight. The system was encased in a protective and leak-proof Zarges box (Fig. 4). The system also had to be mechanically constrained to prevent movement during flight and to ensure it would remain fixed in the event of a plane crash (Supplementary Fig. S1B-C). Thus, the system was bolted to the plane seat rails and mechanical dampeners were added to reduce vibration from the aircraft (Supplementary Fig. S1C). The waterproof Zarges box lid was also interlocked with the fluorescence LED source to prevent dazzling stray light when opened.

We designed a closed microfluidic sample to image on board the parabolic flight and test the systems capability. Yeast cells were adhered to 5-channel microfluidic slides connected to syringes mounted to the syringe pump. The experiment consisted of 6 individual microfluidic sample chips, each consisting of 5 channels on a slide with each channel connected to syringes and waste ports via silicone tubing (Fig. 3B). Each sample system was designed to be used once for each parabola set (Supplementary Fig. S2B) (five parabolas in a parabola set, each lasting 100 s, separated by 90 s and 5 mins between sets (Supplementary Fig. S2A-B). Thus, a 3D printed sample holder system was designed to facilitate fast changeovers during parabola set breaks (Fig. 4(iii), (iv), (v)). A changeover requires one operator to home the syringes while the other operator unclips the old sample rack from the pump, secures it in the holder, before removing and attaching a new sample rack (Supplementary Fig. S4A).We found that our sample holder system allowed a changeover to be performed in under 3 mins, allowing enough time to close the lid ready for the next parabola set (Supplementary Fig. S1A).

## Performance of FlightScope on parabolic flights

**Fig. 5:**
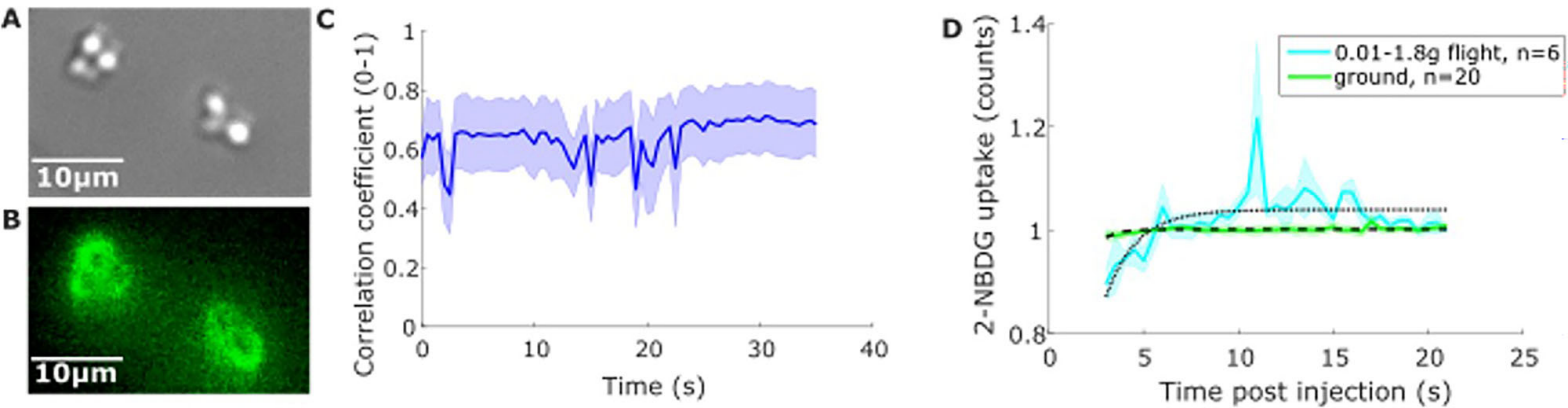
Performance of FlightScope on parabolic flights. **A** Brightfield image of yeast cells and **B** fluorescence image of 2-NBDG uptake by the cells taken by FlightScope on board a parabolic flight. (100 ms exposure, 20% illumination power). **C** Plot of average lateral cell location correlation coefficient for four videos taken in steady 1 g flight. **D** Plot of 2-NBDG uptake by cells when in 0.01–1.8 g flight (cyan) and on the ground (green) with lines of best fit.

Despite the challenges of operating on-board a parabolic flight, Flight-Scope performed well. We were able to obtain similar quality brightfield and fluorescence images of live yeast during the flight (Fig. 5A) as were obtainable on the ground (Fig. 1C–F). We also characterised Flight-Scope’s tolerance to vibration on board the flight, as before, by quantifying the correlation coefficient between consecutive images (Fig. 5C). Vibration tolerance was similar to our ground tests, with correlation coefficients remaining at between 0.5 and 0.8 during steady state (non-parabolic) flight (Fig. 5C), comparable to ground performance under similar vibration (Fig. 1C, F).

During parabolas and changes in apparent gravity, between microgravity and hypergravity, we detected an xy lateral shift of 8.3 ± 0.3μm at the sample plane. This was easily corrected for during analysis. We performed glucose uptake experiments with 2-NDBG and live yeast as on the ground. We obtained proof-of-principal data of glucose uptake during the shift from microgravity into hypergravity (0.01–1.8 g). The intensity of the fluorescence images taken during 0.01–1.8 g flight was characterised for 0.01–1.8 g flight against that for images taken on the ground and plotted against time post 2-NBDG injection (Fig. 5D). These were fitted with exponential uptake fits^33^ with characteristic uptake times of 0.6 ± 0.2s compared to 1.3 ± 0.3s on the ground. These measurements serve as important proof-of-principal of FlightScope, the need to study cell signal transduction in microgravity and the potential for further microgravity experiments going forward.

## Context and future perspective

FlightScope is a new microscopy and microfluidic platform for microgravity research. We demonstrated FlightScope’s capabilities on board an ESA parabolic flight imaging live yeast cells and using our microfluidic system to expose cells to fluorescent glucose in real time during the gravity changes on the flight. The microscope hardware and software performed well and allowed both brightfield and fluorescence imaging, with minimal influence from vibration, as quantified by cell correlation coefficient which remained between 0.5 and 0.8 (Fig. 5D). We did observe a lateral displacement in xy when moving into altered gravity conditions on the flight of ~8 μm, although we could easily correct for this in our analysis. Our microfluidic system performed well on the flight, enabling us to inject fluorescent glucose and observe glucose uptake during changes from microgravity to hypergravity. Although our flight time did not allow a full program of experiments investigating how cell signalling is impaired in microgravity, our proof-of-principal results with FlightScope open the way for future investigations.

Compared to the other sixteen microscopes used in microgravity and hypergravity environments that we have identified and outlined in Table 1, FlightScope has a unique feature set, optimised for life science research on a parabolic flight, including brightfield and fluorescence microscopy, as well as fully motorised xyz control. FLUMIAS-DEA is one of the few microscopes, like FlightScope, purpose built for low gravity operation and boasts some of these capabilities^11^ but lacks brightfield and microfluidics. None of the other microgravity microscopes to our knowledge have microfluidic capabilities. Microfluidics enable the kind of dynamic experiment we demonstrate here, where cell signalling can be observed in real time during changes in gravity. We demonstrate this imaging glucose uptake of budding yeast but FlightScope could be used to investigate many other cell signalling processes in microgravity, such as insulin signalling in human cells which has shown to be impaired^34^ or cancer cell response to chemotherapy, also shown to be modulated by microgravity^35^. We demonstrated FlightScope with relatively short exposure times which would enable cell swimming or cell Brownian motion to be visualised in future applications.

Future iterations of Flight-Scope could benefit from a high speed autofocus or self-stabilising system to correct for the drift we encountered imaging on the plane. The most recent SQUID microscope platform supports autofocus using a contrast-based method or a laser autofocus. A laser autofocus system could be implemented in Flight-Scope to track the coverslip. Laser-based autofocus works well with dimmer samples and is faster than the traditional image-based autofocus^36^. The laser would not pose a risk of bedazzlement due to the containment of the microscope. Alternatively, autofocusing/stabilisation can be achieved with image based methods such as with an electrically-tuneable lens (ETL)^37^. These ETLs are liquid lenses with a motor bobbin controlling the lens volume to focus the images. Although studies into the effects of gravity changes on the liquid within the lens would be required for implementation in FlightScope. Rapid Autofocus via Pupil-split Image phase Detection (RAPID) autofocuses the image using phase detection and could also be implemented on Flight-Scope^38^. There is also no reason in principle why future FlightScope could not also benefit from super-resolution imaging, although many of these techniques e.g. STORM are highly vibration sensitive.

In total, the cost of FlightScope was below £10k (GBP) with the microscope costing ~£6k, housing and fixings ~£2k and the 5-channel syringe pump system only costing ~£800 (GBP). This makes FlightScope an extremely cost-effective instrument. This was in part facilitated by 3D printing which enabled rapid prototyping of syringe pump components to accelerate design and production of FlightScope. Of the sixteen microscopes outlined in Table 1, only the Image acquisition module^39^ is known to also have 3D printed components in its design.

We have demonstrated FlightScope’s capability for microgravity research on-board a parabolic flight but there are many opportunities for this technology on other microgravity platforms. Current microscopy technology used on board the ISS, FLUMIAS-DEA^11^, is a powerful instrument but its commercial design does not easily allow for new imaging modalities to be incorporated. The advantage of an open design, such as FlightScope, is that researchers around the world can easily build their own modules. FlightScope would need further development to miniaturise it, both in size and weight. Even further miniaturisation would allow FlightScope to be deployed on CubeSats, another cost-effective microgravity platform. To fit within the 10 cm^3^ volume of a CubeSat, it is likely a bespoke objective lens would be required or a design using a microlens array^40^.

Finally, FlightScope lends itself to a multitude of other use cases, particularly unusual environmental conditions. The system is fully contained, keeping samples safe from liquids, dust and wind. Therefore, it could be used in harsh conditions such as deserts and jungles. It is readily transportable in a vehicle with the dampening provided to the microscope ensuring that it can function with travel vibrations. FlightScope is a versatile platform for microscopy and microfluidics in microgravity and other extreme environments.

## Methods

### Microscope design

The main optical train (Fig. 1B) of the microscope includes a 60fps Daheng Imaging MER2-630-60U3M USB camera with Sony IMX178 sensor (2.4 μm pixel size), a Daheng Imaging 75 mm lens tube, an Olympus UPLFLN 40X air objective with focal length 180 mm, 0.49 μm depth of field and 0.75 NA (numerical aperture), a Thorlabs 809 mW 470 nm LED used for fluorescence illumination and an Adafruit DotStar high density 8 × 8 RGB LED pixel matrix grid used for brightfield illumination. The optical train, including the camera, lens tube, fluorescence light source and objective, was mounted to a dampened pillar, while the brightfield matrix was mounted on a separate adjustable height pillar, on a hinged arm to allow access (complete list of parts available in the Supplementary Table 1). Focusing was achieved by movement of the entire optical train in the z-axis by a Nema 8 linearly actuated motor and rail with return spring. A motorized 60 × 60 mm xy stage was mounted separately with 4 pillars to an optical breadboard along with the optical train and brightfield pillars. A console with buttons, rotary knob and joystick is used to control the z-axis and xy stage respectively (Supplementary Fig. S3B(iv)), and a printed circuit board (PCB) for control of the lights and movement through a dedicated laptop to display the microscope’s image and control movements through a custom-built Python-based software, on a laptop running Linux allowing the user to change brightfield and fluorescent illumination intensity, exposure time and video recording length as well as control the motorised stage (Supplementary Fig. S3A). The instructions on Github outline the installation of both the software and the drivers to the camera and the control plates, available form https://github.com/hongquanli/octopi-research. The software runs on Linux Ubuntu 20.04 and does not have specialist hardware requirements. The laptop used to run this software in this project was a Dell Inspiron 15 with a i7-1355U processor, Iris Xe graphics card and 16 GB of RAM.

### Microfluidic system and storage

To deliver the 2-NDBG to the yeast during imaging, a bespoke microfluidic system was designed (Figs. 3A, B and 4(ii)). Microfluidic sample systems consisted of 1 mL syringes connected by female and male luer lock connectors, in turn connected to 6-channel microfluidic chip slides (Ibidi μ-Slide VI 0.4, Germany) with silicone tubing (2 mm inner diameter, 3 mm outer diameter). For waste let out of the slides following glucose injection, a balloon cable-tied around the silicone tubing was used (Fig. 3C).

During the flight campaign, there were a total of 30 microfluidic sample systems, one for each parabola during the flight, in the form of 6 sets of systems. Each system consisting of 5 syringes connected, via the silicone tubing, to 5 channels in a sample slide. All samples needed to be secured to prevent the syringes from prematurely injecting during the changes in gravity. The storage system for the syringe panels used a locking mechanism; panels were stored vertically with the syringe plungers pointed towards the wall of the box (to prevent experimenters accidentally knocking them) (Fig. 4(iii)) and right red frame). Each storage unit can hold two panels. The units have a 40×40 extrusion frame with 3D printed part attached. The slides were stored vertically inside a protective 3D-printed housing (Fig. 4 left red frame); these were attached to the floor of the Zarges box with Dual Lock with space for the tubes between the slide holder and syringe storage unit (Fig. 4).

The syringe pumps are intended to be used in a bank of five pumps (although the system can be adapted to handle more). The experiment demanded the syringe pumps be independently controlled as a fresh syringe was required for each parabola (for 6 sets of 5 parabolas). The five syringes are attached to a panel so all five syringes can be removed at once (Fig. 3C), this was necessary to meet the short changeover time of 5 minutes between parabola sets.

### Syringe pump design

The syringe pump was designed with modularity for easy part replacement. Each pump module (Fig. 3B) facilitates substitution in case of failures. The design accommodates the addition of extra pumps, with our experiment utilizing five modules. Made from standard parts, the frame is 3D-printed, the linear motion that creates the pushing force on the syringe plunger is generated by a Nema 17 stepper motor coupled with an M10 threaded rod (chosen over a traditional lead screw to minimise cost and maximise the accessibility for build), a captive nut is concealed within the carriage, the carriage moves along two ground rods. The fine adjustments can be made to the plunger pusher by twisting the bolt to meet the plunger without prematurely injecting the syringe.

The syringe pump components were fabricated using a Flashforge Guider 2 3D printer with a 0.4 mm diameter nozzle. Printing parameters were adjusted to ensure optimal printing time and quality, including a layer height of 0.2 mm, four perimeters wall thickness, five solid layers at the top and bottom, and an infill density of 35%. PLA material was selected for printing as it is readily available and easy to work with.

A bespoke control system was built to independently drive the five syringe pumps used during the experiment. This system consisted of five open-loop stepper motor driver boards, five limit switches, several panel-mount inputs, and a display, all wired to an Arduino Uno flashed with a custom control script. Supplementary Fig. S4E provides a simplified block diagram of the control system components and wiring. Supplementary Fig. S4C provides a top-down view of the components wired together within the control enclosure. Supplementary Fig. S4D shows the panel-mount connectors used to interface the control enclosure electronics with the external pump motors and limit switches, a 24Vdc power supply, and a monitoring computer/5 V power supply.

As each syringe pump had to be operated independently, the stepper motors used in each pump were driven by separate open-loop driver boards. Each of these motor driver boards were provided separate STEP (sometimes referred to as PULSE) and DIRECTION signals (Supplementary Fig. S4F) that were generated by an Arduino Uno flashed with a custom control script. Limit switches attached to each syringe pump were wired to this Arduino, and their states (Open or Closed) were used during individual motor homing. The state of each limit switch was also used throughout pump operation to prevent the carriage from crashing and damaging the pump in the event of misuse (overdriving pumps, for instance). The STEP and DIR signals discussed here were generated by the Arduino based on user input and the states of these limit switches.

An input panel consisting of four push buttons, a motor selector knob, and a display was provided to allow users to control the system. This control panel is shown in Supplementary Fig. S4A, and the connections made to the Arduino Uno within the control enclosure (Supplementary Fig. S4B).

A motor selector knob was provided to allow users to select a syringe pump for control, and four push buttons were supplied to trigger different actions: initiating motor homing, jogging, or dosing with a predefined volume of glucose (Supplementary Fig. S4A). A 16 × 2 character LCD was included in the control panel to give the user succinct pump operation feedback, ensuring the correct operations were performed during testing. A 24 V cut-off switch was provided on this control panel to enable quick power isolation in case of motor stalls or potential user injury caused by equipment misuse. Full design available here https://github.com/Alexander-Stokes/Flight-scope.

### Experiment containment and plane interface

There were several constraints to experimental operation during a parabolic flight set out by Novespace, the company running the flight campaign, to ensure the safety of everyone on-board the flight.

The experiment was fixed within a 750x550x580mm Zarges box to prevent liquid leakage during flight and to supply a rigid structure and a dark space for fluorescent imaging, adhering to liquid and laser containment safety guidelines (Supplementary Fig. S1B(i)). The leak-proof capability of the system was tested by filling the Zarges box with approximately 50 litres of water (prior to fitting the electronics). Further leak-proof measures included sealing the fluidic loops at both ends and covering electronics in silicone to prevent liquid causing a short circuit within the box. All electrical components were scrutinised by Novespace to check that the wire gages were thick enough to prevent burnout causing a fire risk to the aircraft, as well as checking there were sufficient current limiters and fuses within the components preventing a current surge. The experiments power came from the plane at 230 V AC 50 Hz and had a fuse and emergency stop at the outlet.

All equipment within the box was bolted to a 10 mm aluminium base plate which was fixed to the box through four dampeners to further reduce the effect of vibrations generated by the aircraft. A further two 10 mm aluminium plates (875 × 130 mm) were bolted to the seat rails of the Airbus A300, as the box’s interface with the plane (Supplementary Fig. S1B(iii)) (with laptop, microscope control box and syringe-pump control interface all fitted to the baseplate with Dual Lock (Supplementary Fig. S1B(ii))). This fixation received rigorous evaluation by Novespace to ensure the box was safely and securely attached to the aircraft, all components must withstand a 10 G impact, the forces incurred from a worst-case scenario crash-landing.

### Sample preparation

*Saccharomyces cerevisiae* CEN.PK 113-7D yeast was scraped from a YPD agar dish (10 g/L yeast extract, 20 g/L peptone, 20 g/L agar, 4% glucose (w/v)) and cultured overnight in a 30°C shaking incubator in YNB media (13.8 g/L Yeast Nitrogen Base (YNB), 4% glucose (w/v)). 200 μL of the overnight culture was then pipetted into a morning culture, consisting of the same volumes of media and left in a 30 °C shaking incubator for two hours. Final yeast cultures were taken from this and diluted 1:10 in fresh YNB media. 30$\mu$L of Concanavalin A was pipetted into each channel in each sample slide and left to rest for 15 min followed by washing with 30$\mu$L YNB. 30$\mu$L of sample culture was then pipetted into each channel and left to rest for 15 min. After another YNB wash, the silicone tubing filled with fluorescent glucose analogue, 10,25 or 50 µM 2-NDBG (2-(N-(7-Nitrobenz-2-oxa-1,3-diazol-4-yl) Amino)-2-Deoxyglucose), syringes and waste outlets were attached.

## Supplementary information

Supplementary material

## Data Availability

Full instructions, designs and CAD files to build Flight-Scope are available here: https://github.com/Alexander-Stokes/Flight-scope.

## Code availability

Software to operate Flight-Scope is available here: https://github.com/hongquanli/octopi-research.
