# Supplementary material for: Microscopy with microfluidics in microgravity using FlightScope
Source: NPJ Microgravity. 2025 May 6;11:13. doi: 10.1038/s41526-025-00470-3 (PMC12056092; doi:10.1038/s41526-025-00470-3)
Supplement: Supplementary file 1 — Supplementary material [file 41526_2025_470_MOESM1_ESM.pdf]

Supplementary Material

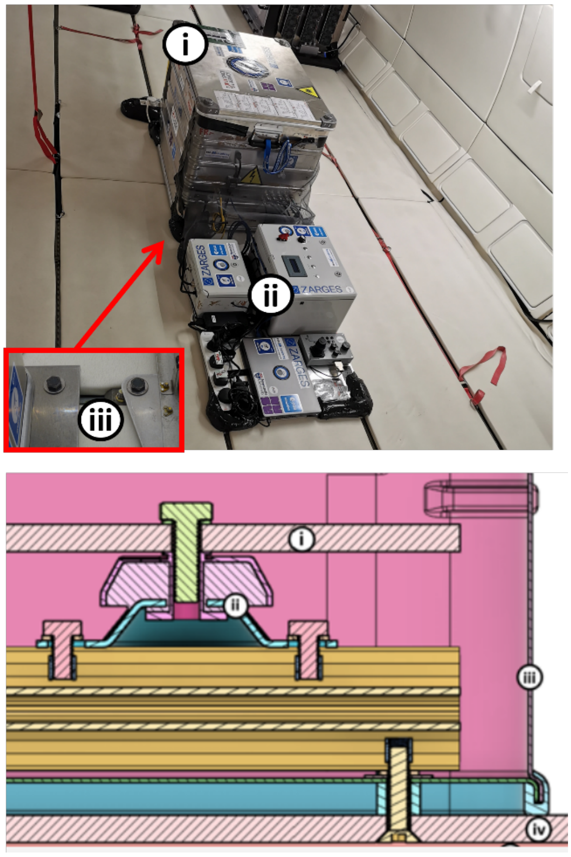

***Figure S1 A**. Top:. Flight-Scope Zarges box (i) and control baseplate (ii) fixed to the Airbus A300 seat-rails with bolts shown in red box (iii). Bottom: Zarges box fixation to seat-rails cross-section (i) inner base plate – microscope and syringe pumps fix to. (ii) dampener. (iii) Zarges box. (iv) external base plate 1/2. (v) seat rails.*

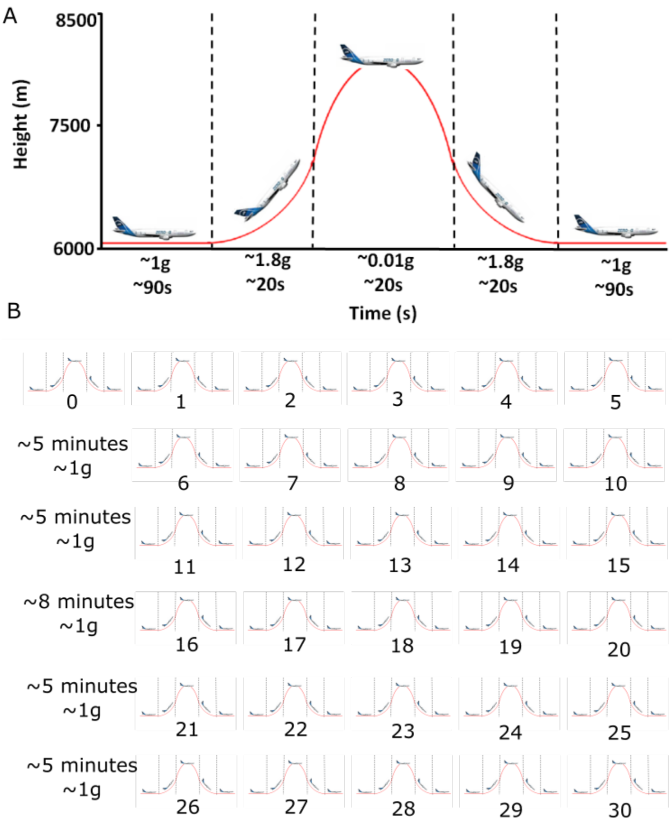

Figure S2 A. *Individual parabolic manoeuvre performed during a Novespace parabolic flight indicating an approximate 20 second interval of ~0.01g sandwiched by two approximate 20 second intervals of ~1.8g. There is an approximate 90 second interval of steady flight, ~1g before and after the varying gravity. At steady flight, the plane flights at approximately 6000m but reaches up to 8500m when performing the parabolic manoeuvre.. .* ***B****. Sets of parabolas separated by approximately 5 minute or 8 minute long breaks. Each flight consisted of 31 parabolic manoeuvres taking on the profile in A.*

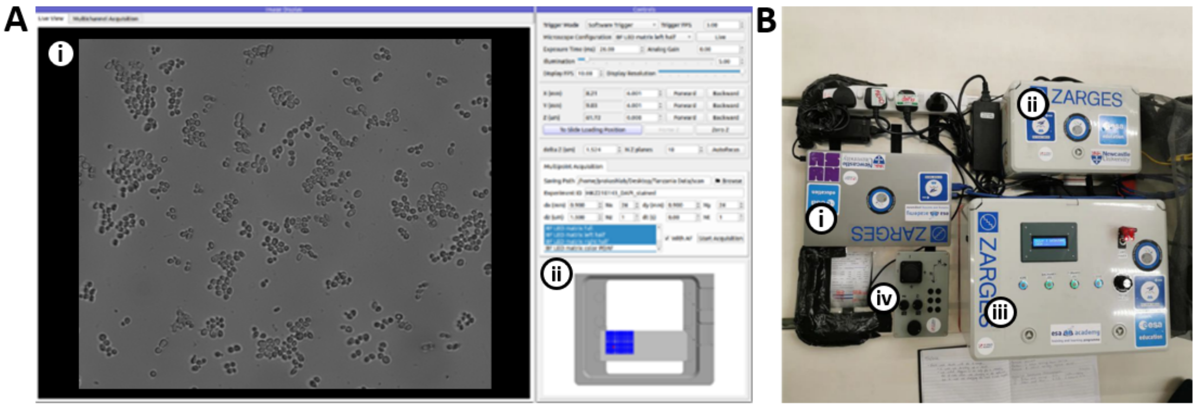

Figure S3 A. *Flight-Scope user interface with as seen on the laptop. (i) field of view live feed from the microscope imaging yeast. (ii) microscope and stage computer control and settings, and map of the microscopes field of view relative to the stage.* ***B****. Control base plate layout (i) laptop. (ii) microscope PCB. (iii) syringe pump control box. (iv) xy stage joystick and z axis dial controls.*

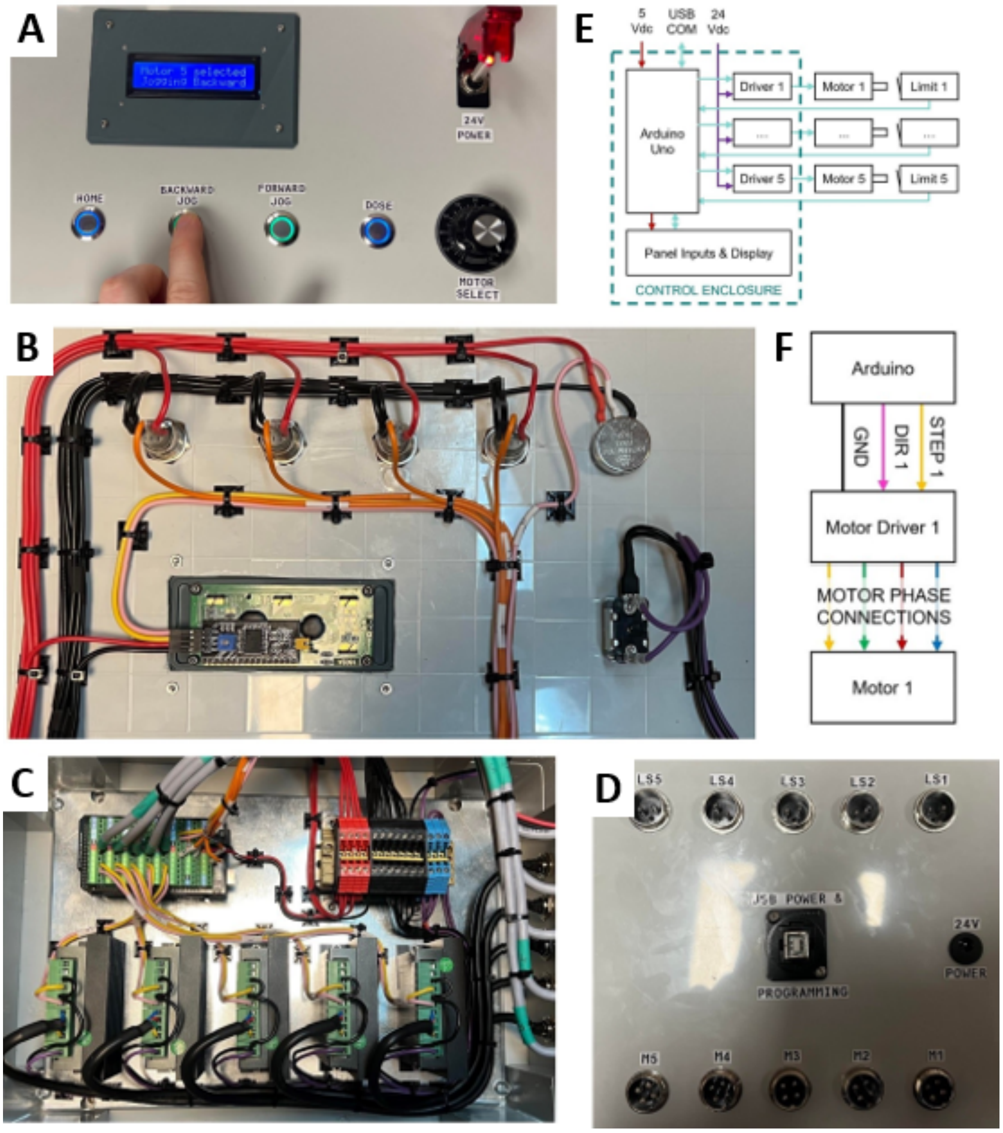

Figure S4 A. *Syringe pump control panel.* ***B.*** *Wiring within the syringe pump control box.* ***C****. motor drivers and Arduino wired together inside the control box.* ***D****. motor, limit switch, power, and USB communication connections on the control box exterior.* ***E.*** *Simplified schematic of component connections and communications.* ***F****. simplified schematic of the connections between each motor, its controller, and the Arduino.*

Supplementary Table S1. Bill of materials

Microscope and controls

Part	Vendor	Code	Quantity	Total cost (£)
0.3" SM1 lens tube	Thorlabs	SM1L03-P5	1	44.61
0.5" SM1 lens tube	Thorlabs	SM1L05-P5	1	46.19
Mounted LED - 470 nm	Thorlabs	M470L5	1	170.94
Adjustable lens tube	Thorlabs	SM1V05	1	23.37
Extra thick retaining ring	Thorlabs	SM1RRC	1	8.57
SM1 to M12 x 0.5 Lens Cell Adapter	Thorlabs	S1TM12	1	18.72
Lens tube sleeve coupler	Thorlabs	SM1CPL10	1	28.28
Kinematic Mirror Mount	Thorlabs	KCB1C	1	110.47
Ø1" Broadband Dielectric Mirror	Thorlabs	BB1-E02	1	58.01
Cage Assembly Rod, 1" Long, Ø6 mm, 4 Pack	Thorlabs	ER1-P4	2	29.66
N-BK7 Best Form Lens, Ø1", f = 75 mm, ARC: 350-700 nm	Thorlabs	LBF254-075-A	1	43.88
Cage Cube with Dichroic Filter Mount	Thorlabs	CM1-DCH	1	134.77
lens retaining ring	Thorlabs	SM1RR	3	10.44
SM1-RMS adapter	Thorlabs	SM1A3	1	13.88
Lens tube coupler	Thorlabs	SM1T4	1	20.76
End cap	Thorlabs	SM1CP2	1	14.48
M30.5-SM1 adapter	Thorlabs	SM1A15	1	17.45
M27-SM1 adapter	Thorlabs	SM1A36	1	16.4
Thorlabs SM1TC lens tube clamp	Thorlabs	SM1TC	1	35.15
Adjustable 1.5" post mounting clamp	Thorlabs	C1511	1	56.67
Condenser Lens	Thorlabs	ACL2520U-DG6-A	1	23.71
Cage Plate for the condenser	Thorlabs	CP33-SM1	2	14.32
Cage Plate for the condenser f = 30 mm	Thorlabs	CP33T	1	18.78
Cage rods - 2"	Thorlabs	ER2-P4	2	35.82
90° flip mount for	Thorlabs	FM90	1	68.21

breadboard 10" x 12"	Thorlabs	MB1012	1	109.8
horizental 0.5" post for the LED matrix	Thorlabs	TR4	1	4.76
4-Pin Female Mating Connector for Mounted LEDs	Thorlabs	CON8ML-4	1	25.58
Ø1.5" Dynamically Damped Post, 10" Long	Thorlabs	DP10A/M	1	169.28
Right-Angle End Clamp	Thorlabs	RA180/M	1	8.81
Ø12.7 mm Optical PostL = 250 mm	Thorlabs	TR250/M	1	7.69
Ø1/2" Optical Post L = 6"	Thorlabs	TR6	1	5.78
Universal Post Holder	Thorlabs	UPH6	1	29.94
Clamping Fork 5 Pack	Thorlabs	CF038-P5	1	33.42
Olympus UPLFLN 40X objective	Edmund	#86-860	1	1215.5
XY stage + cable	Heidstar	HDS-U-XY6060SN	1	1669.70
Z stage + cable	Heidstar	HDS-S-Z6SN-STF	1	882.43
Controller + LED driver	Heidstar	N/A	1	755.04
LED matrix	Heidstar	N/A	1	144.26
Joystick + cable	Heidstar	N/A	1	700.44
Monochrome camera + trigger + USB	Heidstar	MER2-630-60U3M	1	205.14
50mm imaging lens	Heidstar	MVL-F5024M-10MP	1	106.08
AlexaFluor 488 filter cube	N/A	N/A	1	425.38
Studded Pedestal Base Adapter	Thorlabs	BE1/M	4	32.48
Pillar post m6 taps L = 50mm	Thorlabs	RS50/M	4	76.84
Pillar post m6 taps L = 75mm	Thorlabs	RS75/M	4	83.84
adapter sm1 to RMS	Thorlabs	SM1A36	1	13.88
USB 3.0 A Male to A Male Lead, 1m Blue	Farnell	CAC250018	1	3.70
HYLEC IP65 ENCLOSURE	Screwfix	2597G	1	31.65
36 mdr cable	Ebay	mdfly electronics	1	40.92
Linux laptop	Dell	Latitude 5440	1	799.9
			**Total =**	8645.74

Syringe pump and controls

Part	Vendor	Code	Quantity	total cost
TB6600 stepper driver x6	Robotshop	RB-Dfr-727	6	£95.16
PLA Filament	RS	832-0264	1	£30
Linear bearings 8mm ID x10	Robotshop	RB-Sct-1372	10	£42.30
10 mm bearing	RS	441-9969	5	£25.05
limit switches	Robotshop	RB-Tam-71	6	£9.90
Nema stepper motor x3	Amazon	N/A	2	£47.98
Shaft coupler	Robotshop	RB-Sct-1160	6	£36.60
Flexible couplings	NU Workshop	N/A	5	£48.72
M10 threaded rod	NU Workshop	N/A	5	£87.86
screws	Screwfix	N/A	1	£12.49
Wago 5-Way Terminal Block, 32A, Spring Cage Terminals, 24 # 12 AWG, Cable Mount	RS	883-7557	1	£6.79
RS PRO Straight, Panel Mount, Socket to Socket Type B to A 2.0 USB Connector	RS	916-0227	1	£9.67
RS PRO 4 Pole Din Socket, 2A, 100 V ac, Twist Lock, Female, Panel Mount	RS	786-3429	1	£3.38
HYLEC IP65 WEATHERPROOF OUTDOOR ENCLOSURE 300 X 220 X 400MM	Screwfix	6608G	1	£52.84
GOLDSCREW PZ COUNTERSUNK MULTIPURPOSE SCREWS 4 X 25MM 200 PACK	Screwfix	17430	1	£2.69
GOLDSCREW PZ COUNTERSUNK MULTIPURPOSE SCREWS 3 X 25MM 200 PACK	Screwfix	14448	1	£1.99
RS PRO Square Bracket for Top Hat DIN Rail 10PK	RS	467-383	1	£29.24
Weidmuller Red WDU Feed Through Terminal Block, Single level, 2.5mm², ATEX, 800 V 10 PK	RS	779-649	2	£15.86
Weidmuller Blue WDU Feed Through Terminal Block, Single level, 2.5mm², ATEX, 800 V 10PK	RS	425-285	2	£14.40
Weidmuller Black WDU Feed Through Terminal Block, Single level, 2.5mm², ATEX, 800 V 10PK	RS	779-627	2	£15.86
Weidmuller W ATEX End Cover for DIN Rail Terminal Blocks 5PK	RS	425-291	3	£4.53

Weidmuller EW ATEX End Stop for DIN Rail Terminal Blocks	RS	425-314	1	£6.74
Weidmuller WQV Jumper Bar for DIN Rail Terminal Blocks	RS	425-336	3	£47.82
Weidmuller ATEX 2 Way Screw Down WPE 2.5, 60mm Length 30 → 12 AWG	RS	193-124	1	£11.74
RS PRO Steel Slotted Din Rail, Top Hat Compatible, 1m x 35mm x 7.5mm	RS	467-416	1	£19.94
6 core cable 100m reel, unscreened white PVC	RS	8200182	1	£59.82
Arduino mega controller	RS	769-7412	1	£35.70
adjustable spanner	Screwfix	86962	1	£9.99
long nut	Screwfix	728GX	1	£1.79
			**Total =**	£786.85

Experimental containment and fitting

**Part**	**Vendor**	**Code**	**Quantity**	**total cost**
K 470 universal container, IP 65	ZARGES	366218	1	£700
Anti Vibration Mounts	WDS	WDS 719-20540	4	30.68
Toggle latch	WDS	4202-201K2	12	21.72
Machined aluminium parts	N/A	N/A	1	200
Aluminium extrusion and brackets	N/A	N/A	1	227.87
Aluminium plates	metal supermarkets	N/A	1	499.1
Dual lock	Amazon	N/A	1	14.9
balloons	Amazon	N/A	1	3.89
Noga HSS RB 1000 Deburring Tool for Deburring	RS	339-4336	1	12.03
RS PRO IEC C7 Socket to BS 1363 UK Plug Power Cord, 1.5m	RS	452-669	2	19.88
RS PRO Pozidriv Countersunk A4 316 Stainless Steel Machine Screws DIN 965, M4x16mm	RS	158-3629	1	9.23
Plain Stainless Steel, Hex Bolt, M10 x 35mm	RS	797-6282	1	23.39
RS PRO Black, Self-Colour Steel Hex Socket Countersunk Screw, DIN 7991, M8 x 40mm	RS	822-9149	1	11.12

RS PRO Black Nylon Cable Tie, 100mm x 2.5 mm	RS	233-455	2	5.38
RS PRO Self Adhesive Black Cable Tie Mount 12.5 mm x 12.5mm, 3.2mm Max. Cable Tie Width	RS	811-1723	4	32.2
3M Dual Lock™ SJ3540 Black Hook & Loop Tape, 25mm x 2.5m	RS	163-4737	1	33.51
10m Magnetic Tape, Adhesive Back, 0.75mm Thickness	RS	297-9116	1	14.96
DETA RUBBER GASKETS 68MM 10 PACK (9076J)	Screwfix	9076J	1	1.43
NO-NONSENSE GENERAL-PURPOSE SILICONE CLEAR 310ML	Screwfix	35887	1	4.59
silicone tubing	Amazon	N/A	1	55.18
Mosquito Net	N/A	N/A	1	10.99
Big cable ties	N/A	N/A	1	7.9
PLA Filament	RS	832-0264	1	£30
			**Total =**	£1970
